# Clinical Utility and Implementation of Pharmacogenomics for the Personalisation of Antipsychotic Treatments

**DOI:** 10.3390/pharmaceutics16020244

**Published:** 2024-02-07

**Authors:** Marta Hernandez, Natalia Cullell, Marc Cendros, Alexandre Serra-Llovich, Maria J. Arranz

**Affiliations:** 1 PHAGEX Research Group, University Ramon Llull, 08022 Barcelona, Spain; martahh1@blanquerna.url.edu; 2 School of Health Sciences Blanquerna, University Ramon Llull, 08022 Barcelona, Spain; 3 Fundació Docència i Recerca Mútua Terrassa, 08221 Terrassa, Spain; ncullell@mutuaterrassa.cat (N.C.); alexserra@mutuaterrassa.cat (A.S.-L.); 4 Department of Neurology, Hospital Universitari Mútua Terrassa, 08221 Terrassa, Spain; 5 EUGENOMIC Genómica y Farmacogenética, 08029 Barcelona, Spain; mcendros@gmail.com

**Keywords:** pharmacogenetics, pharmacogenomics, antipsychotics, genetic testing, clinical implementation

## Abstract

Decades of pharmacogenetic research have revealed genetic biomarkers of clinical response to antipsychotics. Genetic variants in antipsychotic targets, dopamine and serotonin receptors in particular, and in metabolic enzymes have been associated with the efficacy and toxicity of antipsychotic treatments. However, genetic prediction of antipsychotic response based on these biomarkers is far from accurate. Despite the clinical validity of these findings, the clinical utility remains unclear. Nevertheless, genetic information on CYP metabolic enzymes responsible for the biotransformation of most commercially available antipsychotics has proven to be effective for the personalisation of clinical dosing, resulting in a reduction of induced side effects and in an increase in efficacy. However, pharmacogenetic information is rarely used in psychiatric settings as a prescription aid. Lack of studies on cost-effectiveness, absence of clinical guidelines based on pharmacogenetic biomarkers for several commonly used antipsychotics, the cost of genetic testing and the delay in results delivery hamper the implementation of pharmacogenetic interventions in clinical settings. This narrative review will comment on the existing pharmacogenetic information, the clinical utility of pharmacogenetic findings, and their current and future implementations.

## 1. Introduction

Antipsychotic drugs are the mainstay treatment for psychotic symptoms of schizophrenia and other severe mental disorders. However, about 30–50% of people receiving antipsychotics do not respond adequately and/or develop severe and long-lasting side effects. The reasons behind treatment failure and adverse reactions are difficult to discern as the precise mechanism of action of antipsychotic drugs is still unclear. Clinical and environmental factors [1] as well as genetic factors [2] have been proposed as contributors to variability in response to antipsychotic treatment.

Pharmacogenetic and pharmacogenomic research investigate the genetic contribution to response variability at individual loci and genome-wise level, respectively. The main aims of pharmacogenetic and pharmacogenomic investigations are the unravelling of the mechanism of action of antipsychotics and the identification of response predictors that could help in the improvement and personalisation of treatment. Decades of pharmacogenetic research have revealed individual genetic biomarkers of clinical response to antipsychotics [3]. However, prediction of antipsychotic response based on genetic biomarkers is far from accurate. Despite the clinical validity of several pharmacogenetic findings, the clinical utility remains unclear. Nevertheless, genetic information on metabolic enzymes involved in the biotransformation of most commercially available antipsychotics has proven to be useful for the reduction of treatment induced side effects [4,5]. At present, the implementation of pharmacogenetic interventions in psychiatric settings is very limited, and further proof of their clinical utility is required. Pharmacogenomic research conducted in the last decade has advanced our knowledge on the mechanism of action of antipsychotic drugs by confirming the therapeutic value of known targets [2,6]. Further research into genomic and epigenomic factors contributing to response may help to improve the accuracy and clinical applicability of genetic information in psychiatry.

The aim of this narrative review is to summarise the knowledge on the pharmacogenetics and pharmacogenomics of antipsychotic drugs and to give an overview of their current clinical applications.

## 2. Methods

### Literature Review

**Figure 1:**
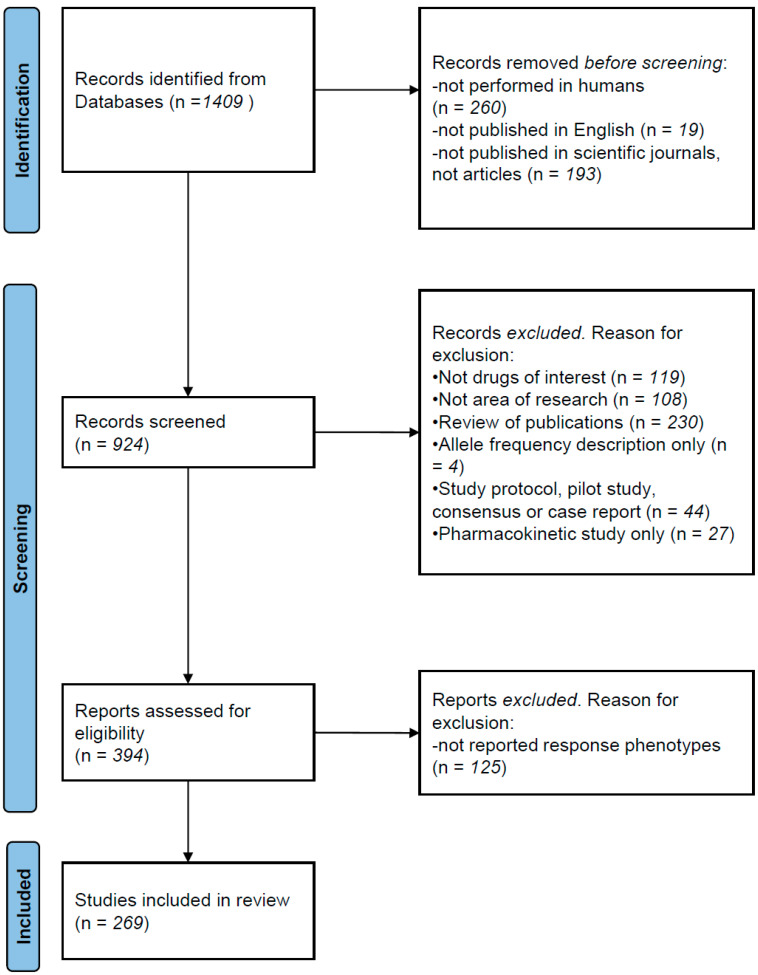
PRISMA description of literature search.

The recommendations of the Preferred Reporting Items for Reviews and Meta-Analyses statement (PRISMA, 2021) were followed for the literature search. We searched the ISI Web of Science using the Boolean method with the following terms: (1) “antipsychotic” OR “chlorpromazine” OR “droperidol” OR “fluphenazine” OR “haloperidol” OR “loxapine” OR “perphenazine” OR “pimozide” OR “prochlorperazine” OR “thioridazine” OR “thiothixene” OR “trifluoperazine” OR “aripiprazole” OR “asenapine” OR “brespiprazole” OR “cariprazine” OR “clozapine” OR “iloperidone” OR “lurasidone” OR “olanzapine” OR “paliperidone” OR “quetiapine” OR “risperidone” OR “ziprasidone”; (2) “pharmacogenetic” OR “pharmacogenetics OR “pharmacogenomic OR pharmacogenomics”. We restricted the search from January 2010 to November 2023 (see Figure 1 for description of process). The references and discussions of all pooled articles were carefully scanned for additional publications. Before screening, we excluded studies (1) not performed in humans; (2) not published in English; and (3) not published in scientific journals. A total of 1406 studies were identified. Articles were excluded from this revision for the following reasons: (1) not drug of interest (not antipsychotics); (2) other areas of research (Alzheimer disease, depression, implementation of informatic tools, etc.); (3) not original new data (reviews of publications); (4) case-report studies; (5) studies with exclusive pharmacokinetic design and outcomes; (6) studies with not reported response phenotype. A total of 269 relevant papers were identified that reported original pharmacogenetic or pharmacogenomic results on a variety of antipsychotic response phenotypes (influence of genetic variants on treatment response, adverse reactions and/or drug clearance). Several limitations to this review need to be considered. Firstly, it is likely that the most recent publications that are not yet listed in search engines, as well as other relevant papers that did not fit our search terms, have been missed. Secondly, many important findings regarding the pharmacogenetics of antipsychotics were published before the 2010 cut-off, although most clinically relevant findings have been replicated in more recent years. Furthermore, there is publication bias in favour of studies with positive findings, and negative or contradictory findings may be underrepresented. Despite these limitations, the next sections will summarise the most significant findings in recent years and will discuss the current applicability of those findings for the personalisation of antipsychotic treatment.

## 3. Pharmacogenetic Studies

Pharmacogenetic or candidate-gene studies are mainly focussed on a limited number of genes selected from the kinetic or dynamic profile of antipsychotic drugs, although genetic variants determining response may also be related to disease pathology. Findings of candidate gene studies are rarely universally replicated. Limited samples sizes, differences in the duration and type of treatment, patient ethnicity, etc., may hinder the replication of results [2]. Furthermore, treatment response and clinical outcome are difficult to measure and may complicate the interpretation of results and explain discrepancies between studies. Replication of candidate-gene findings in independent studies and meta-analyses of published results are necessary to confirm and clarify the strength of the associations. A summary of candidate-gene studies reporting significant findings published in the last decade can be found in Table 1. Older papers with significant results reported or included in later publications have been excluded. While not fully comprehensive, this section will discuss the most relevant and clinically valuable findings to date.

### 3.1. Pharmacogenetic Associations with Treatment Response

Given the multitarget profile of most antipsychotics, it is not surprising that many genes related to neurotransmitter receptors and transporters have been associated with variability in treatment response. Dopamine and serotonin neurotransmitter pathways have been thoroughly investigated for possible pharmacogenetic markers [2]. Although the mechanism of action of antipsychotics is not fully understood, it is known that dopamine blockade is directly associated with antipsychotic efficacy [2]. Numerous studies have described associations between genetic variants in dopamine receptors and treatment response [7,8,9,10], with the *DRD2* rs1799732 (−141C Ins/Del) deletion significantly associated with poorer antipsychotic drug response (OR = 0.65 for the Ins allele in meta-analyses) [2]. Although initially a non-significant trend of association of the *DRD3* Ser allele with poor clozapine response was observed, a later meta-analysis [11] confirmed the association of the 9Gly allele with good response in Caucasians, but not in other ethnic groups (OR = 0.72; *p* = 0.002). Genetic variants in serotonin receptors have also been associated with treatment response, particularly to second-generation antipsychotics. Recent studies confirm the association, with *HTR2A* rs6313 T allele carriers showing generally better improvement than non-carriers [2,12]. *HTR3* variants have been associated with clozapine response in Indian patients [13], although the strength of the association varied according to the response phenotype investigated, illustrating the difficulties in assessing clinical outcome. The associations of *HTR2A* and *HTR3* variants with clozapine response have also been confirmed via meta-analyses (OR = 0.68 and 0.45, respectively) [14]. Histaminic receptors, a target of several second-generation antipsychotics (SGA) of unclear therapeutic validity, may contain genetic variants influencing response [15,16,17]. Other genes not directly targeted by antipsychotics but involved in monoamine pathways and/or mental disorders have been associated with response. The *COMT* rs4680 variant (Val158Met), associated with reduced cortical dopamine [18], has been associated with response to second-generation antipsychotics [19,20]. A recent meta-analysis confirmed the strength of this association (Z = 6.709, *p* = 9.8 × 10^−12^) [21]. Variants of the *BDNF* gene were associated with risperidone and clozapine response [22] and with treatment resistance [23].

### 3.2. Pharmacogenetic Associations with Antipsychotic-Induced Adverse Reactions

Antipsychotic treatment induces severe side effects such as extra-pyramidal side effects (EPS) and weight gain (WG). There is strong evidence on the contribution of genetic factors to the development of adverse reactions during antipsychotic treatment. The influence of functional polymorphisms in metabolic enzymes on the clearance of antipsychotic drugs has been proven in numerous studies [2,4,24]. Cytochrome P450 (CYP) functional variants associated with slower (poor metabolisers) or faster (rapid or ultrarapid metabolisers) metabolic rates are known to significantly contribute to antipsychotic efficacy and toxicity [2,25,26]. More robust data have been obtained for functional variants in CYP2D6 and CYP2C19 enzymes. *CYP1A2* and *CYP2D6* functional variants have been associated with tardive dyskinesia (TD) [27,28]. Patients with poor or intermediate CYP2D6 metaboliser phenotypes are at greater risk for risperidone and haloperidol adverse reactions [29,30]. Currently, genetic variants of CYP enzymes are the most used biomarkers to personalise psychiatric treatment, as will be discussed later. Several studies have found a relationship between oxidative stress and antipsychotic-induced TD based on genes involved in the antioxidant defence mechanism, dopamine turnover and metabolism [31]. *DRD2* polymorphisms have been related to akathisia and other movement disorders [32]. Expression of the *HSPG2* gene is altered by antipsychotic treatment and has been associated with movement disorders in animal studies. Numerous studies have investigated its association with EPS, and a meta-analysis found a significant association between the *HSPG2* rs2445142 variant and TD [33]. Regarding weight gain, variants in *HTR2C*, *LEP*, and *MC4R* genes have been confirmed as genetic markers with moderate genetic effects (ORs = 1.47–1.96) [34,35]. HTR2C is involved in the regulation of appetite, and its contribution to weight gain and body mass index during antipsychotic treatment has been thoroughly investigated.

The *HTR2C* rs3813929 (-759-C/T) variant is the most frequently studied single-nucleotide polymorphism and one of the most strongly associated with antipsychotic-induced WG, with the rs3813929-T allele predicting less WG and body mass index (BMI) [36,37,38,39]. Gene variants in *LEP* and *LEPR*, two genes involved in the regulation of lipid metabolism, have been related to antipsychotic-induced WG and short-term dyslipidaemia [40,41]. A recent meta-analysis including 12 studies found no association between the *LEP* rs7799039 polymorphism and WG, although a significant association was found within a subgroup of first-episode schizophrenia patients (OR = 2.32, *p* = 0.0009) [42]. One of the most robust findings is the association of genetic variants in the *MC4R* gene with antipsychotic-induced WG. The *MC4R* was previously associated with obesity in the general population, adding plausibility to the finding, which has been replicated in later studies [43,44]. Finally, an *HLA-DQB1* variant (6672G/C, rs113332494) was robustly associated with clozapine-induced agranulocytosis [45]. The association of *HLA* variants with antipsychotic-induced agranulocytosis was later corroborated in genome-wide association studies [46,47]. The strength of the association increases when patients’ ancestry is considered (OR = 16.67–53.70) [45].

Other findings reported in single studies (see Table 1) require confirmation in independent samples to determine the validity and strength of the associations. In general, the strength of the reported genetic associations is moderate (odds ratios rarely >3). This is not surprising given the multitarget profile of most currently used antipsychotics and the polygenic characteristics of treatment response. However, this suggests that the clinical utility of these findings is limited, as discussed later.

## 4. Pharmacogenomic Studies

As we have seen in the previous section, candidate-gene studies have succeeded in the identification of treatment response markers, some of which are currently in use for the personalisation of psychiatric medication. However, pharmacogenetic studies are based on existing knowledge on the selection of candidate genes, and their contribution to the understanding of the mechanism of action of antipsychotics is limited. Advances in technology have allowed thorough genomic investigations of disorders and treatment outcomes. No previous knowledge is required as the whole genome is investigated. However, genomic studies on antipsychotics have been marred by limited sample sizes that increase the probability of false-negative and -positive findings. Most pharmacogenomic studies on antipsychotics have been conducted with moderate sample sizes (*n* < 1000). Several studies included replication in independent samples to confirm their findings. Table 2 includes a summary of genome-wide and whole exome sequencing studies on antipsychotic response and their side effects published during the last decade.

A BeadChip study of 6789 SNPs conducted by Drago et al. [48] found association between response to SGA and first-generation antipsychotics (FGA) and two *ARID5B* SNPs, a gene involved in autoimmune disorders that are often found in schizophrenia patients. A study investigating variants in 1996 genes on patients treated with olanzapine and risperidone also found associations of SNPs in chromosome 6, a region where the *HLA* gene is located, with treatment response, reinforcing the evidence on the influence of genetic variant in immunologic pathways in clinical outcomes [49]. A genome-wide association study (GWAS) on Indian patients also found a variant (rs4795893) in a gene involved in pro-inflammatory response (*CCL2*) as well as a variant in a gene involved in mental disorders (*NRG1* rs13250975), amongst others, associated with response to SGA and FGA [50]. Two genes not previously associated with treatment response or mental disorders, *MYOTB* and *MTRR*, were found to contain variants associated with antipsychotic response using whole exome sequencing (WES) [51]. This strategy also identified polymorphisms in the genes *NMDA* and *AMPA*, involved in glutamatergic pathways, associated with response [52]. Alterations in glutamatergic pathways have long been associated with the aetiology of schizophrenia, although no clear association with treatment response has been described to date. Interestingly, a recent GWAS also found association between genetic variants in the glutamatergic *GRM7* gene and treatment response [53]. The same study identified novel associations of *GPR12* and *MAP2K3*, two genes involved in glutamatergic pathways, with response using WES techniques, although no finding was replicated by both strategies. Finally, a sequencing study of 143 genes revealed statistically significant association between polymorphisms in *DRD3* (rs342026) and in a neighbouring gene (*PLK5* rs1261027) with olanzapine response, confirming the findings of candidate-gene studies [54].

Regarding adverse reactions, Tanaka et al. described an association between a variant (rs6977820) in the gene *DPP6* and treatment-induced TD, although the biological relation of this gene with the adverse reaction is not clear [55]. Alkelai et al. [56] performed WES in patients treated chronically with antipsychotics and found a significant enrichment of *RIMS2* variants associated with TD. The authors report that the expression of this gene was observed to be increased in the brains of schizophrenic patients in a previous study. One of the most robust pharmacogenetic findings to date is the association of genetic variants in the melanocortin 4 receptor (*MC4R*) with antipsychotic-induced WG, an association that has been confirmed in GWAS and candidate-gene studies (see Table 1). Several novel associations with antipsychotic-induced WG have been reported in subsequent genomic studies but have not yet been replicated and require further study [57,58,59]. Two GWAS studies have confirmed the previously reported association between genetic variants in the HLA complex and clozapine-induced agranulocytosis [46,47]. Furthermore, a GWAS study described a mutation in the *AKCR1* gene, rs2814778, previously associated with lower neutrophil counts, associated with clozapine-induced neutropenia [60]. Finally, a relatively large GWAS study identified variants in two genes, *ATAD3B* and *SKIL*, related to antipsychotic-induced QTc interval change. The *ATAD3B* gene plays a role in inflammatory pathology, whereas alterations in the expression of the *SKIL* gene may be related to the aetiology of schizophrenia [61]. Despite their moderate power, these genomic studies have confirmed the importance of genes involved in inflammatory response and in glutamatergic pathways on treatment variability in the development of antipsychotic-induced side effects. However, some of the findings are difficult to interpret given the modest sample sizes and the difficulty in determining the interaction with other environmental and clinical factors involved in response. Independent candidate-gene studies may be required to confirm the validity of findings of underpowered GWAS and WES studies.

Aside from alterations in the DNA sequence, other genomic factors such as transcription regulation and epigenomic events may play a role in antipsychotic response variability. The study of gene expression and epigenetic changes may provide information on gene × environment interactions and yield more accurate predictions of clinical outcomes. Animal studies have shown that risperidone treatment induces gene expression changes associated with susceptibility to EPS, with the involvement of the mTOR pathway [62]. A study suggested that the basal expression of four genes can predict antipsychotic response. However, the estimation was based on a limited number of patients (*n* = 30) and needs replication in independent studies [63]. Several studies have provided evidence suggesting that antipsychotic medications modulate DNA methylation and that these alterations may influence treatment response [64,65]. Animal studies have shown that methylation levels of dopaminergic genes are altered by olanzapine treatment [64]. DNA methylation changes have been correlated with clinical improvement in MDD patients treated with antidepressants, and similar effects may occur in antipsychotic-treated patients [66]. Human studies have shown that global methylation levels are decreased in schizophrenia patients in comparison with controls, and antipsychotic treatment may partially explain the decrease [65]. Methylation within a recognition sequence for HES transcriptional repressors was found to correlate with clinical improvement after antipsychotic treatment [67]. Research on the interaction of genomic events and environmental factors on antipsychotic response is ongoing despite the difficulty in obtaining large, detailed cohorts for study.

## 5. Clinical Utility of Findings

The two previous sections have reviewed numerous studies that have identified genetic markers of variability in antipsychotic treatment. Pharmacogenetic and pharmacogenomic studies have succeeded in validating antipsychotic targets of therapeutic value as well as provided novel information highlighting new areas of putative therapeutic value. However, despite many of these findings having been confirmed and classified as true pharmacogenetic markers, their clinical utility for the personalisation of antipsychotic treatment is not clear. Even with the strong evidence confirming the association of dopaminergic and serotonergic variants and treatment response, their moderate to low genetic effects indicate that their contribution is not determinant on antipsychotic treatment outcome, and that taken individually, they could not predict response accurately. Similarly, *HT2RC* and *MC4R* associations with antipsychotic-induced WG have been confirmed in independent studies; however, they have no clear clinical utility to discriminate between different antipsychotics despite their varied pharmacological profiles. The strong association of genetic variants in the HLA complex with clozapine-induced agranulocytosis may help to predict if the patient has a high or low risk of developing severe neutropenia [68], but the relatively modest sensitivity of these markers (31–41%) [46,47] does not reach the 50% threshold for clinical applicability [69], although the addition of patient’s ancestry in the prediction algorithm may increase the clinical value [45]. Algorithm combinations of information in several genes and polygenic risk scores have been proposed to improve prediction of antipsychotic response and induced side effects. An algorithm combination of four SNPs in genes involved in mTOR regulation had a 66% accuracy to predict EPS in a sample of *n* = 356 antipsychotic-treated patients [70]. A panel of genetic variants in 15 genes predicted efficacy in 60% of cases and medication tolerability in 71% of the *n* = 352 patients included in the study [71]. Patients with higher polygenic risk scores for schizophrenia tended to have less improvement with antipsychotic drug treatment [72]. Interestingly, polygenic risk scores for schizophrenia, diabetes and BMI were also associated with AIGW [73]. Although these strategies may be useful as prognostic tools, their clinical utility for the selection of treatment remains unclear. Furthermore, most of the pharmacogenetic markers in drug targets may not help to discriminate between antipsychotic treatments given their multitarget profile and the low-to-moderate genetic effects observed in the reported associations.

While pharmacogenetic and pharmacogenomic research is still ongoing to improve response prediction and clinical utility, it is likely that the best genetic biomarkers with the greatest effect and clinical utility have already been discovered. Certainly, functional CYP polymorphisms may have both clinical utility and applicability. Functional polymorphisms in CYP enzymes have been clearly associated with the clinical outcome of antipsychotic treatments. The presence of CYP poor-metaboliser variants is strongly associated with increased adverse reactions, whereas ultrarapid-metaboliser variants may contribute to treatment failure and toxicity [2]. Guiding treatment according to CYP phenotypes has been proven useful in children [74]. Furthermore, a recent study showed that long-acting antipsychotics may still be affected by CYP genetic variants [75]. These associations are of a higher magnitude than the ones observed with genetic variants in antipsychotic targets and may offer useful information on appropriate dosing according to the functional variants carried by the patient. In the case of antidepressants, clinical dosing recommendations based on the patient’s genetic profile have already been issued by the Clinical Pharmacogenetics Implementation Consortium (CPIC) [76,77]. Several studies have proven the clinical benefits of using CYP information for the adjustment of clinical doses or selection of alternative treatments when genetic contraindications are observed. Growing evidence suggests that information on CYP1A2, CYP2C19 and CYP2D6 may be useful for the adjustment of clinical doses of antipsychotics [78,79]. In a previous study, we demonstrated that the dose adjustment of the commonly used antipsychotics haloperidol, risperidone, aripiprazole, clozapine and olanzapine according to the genetically determined phenotype of CYP2D6, CYP1A2 and CYP2C19 resulted in a decrease of side-effects [4]. A similar pharmacogenetic intervention for children and adolescents with autism spectrum disorders resistant to treatment resulted in clear improvement in 80% of cases as well as a reduction in health-associated costs [80]. CYP2D6 and CYP2C19 genotyping improved response in addition to clinician’s and patient’s satisfaction in patients given antipsychotics or antidepressants [81]. As further evidence of the clinical and economic benefits emerges, the implementation of CYP information for the improvement of antipsychotic response will surely increase in clinical settings.

Further improvement of pharmacogenetic predictions may be achieved by investigating the interaction between genes, environment, clinical and demographic factors. A recent study suggests that the consideration of pharmacogenetic and phenoconversion data that considers concomitant treatment and other dose-altering environmental factors may increase the accuracy of antipsychotic dose selection [82]. An additional area that merits further research is the possible interaction of pharmacogenetics x age. Older children are more likely to experience EPS than younger children, whereas antipsychotic adverse reactions such as sedation, weight increase and fatigue in particular are more prevalent in children than in adults [83,84]. Despite this evidence, few pharmacogenetic studies have been conducted in children. In summary, antipsychotic selection based on pharmacogenetic findings may be difficult to achieve, although information on pharmacokinetic genes has a real value for the selection of appropriate clinical doses resulting in improved efficacy and less toxicity.

## 6. Implementation of Pharmacogenetics for Antipsychotics

Despite the growing evidence of the clinical benefits, pharmacogenetic information is rarely used in psychiatric settings as a prescription aid. There are several reasons for this: limited evidence on the clinical benefits and cost-effectiveness of pharmacogenetic interventions, few clinical guidelines based on pharmacogenetic biomarkers, and delay in delivery of results, amongst others [2,85].

The clinical utility of pharmacogenetic-guided antidepressant treatment has been proved in several studies Genotyping for key CYP functional polymorphisms and variants in the serotonin transporter gene (*SLC6A4*), the main target of SSRIs, can be used to select antidepressant type and dose with significant improvement in efficacy [79]. A recent study showed that children presenting pharmacogenetic contraindications for the antidepressant treatments they were receiving showed poorer response than those without contraindications [86]. However, evidence on the clinical benefits of pharmacogenetic interventions for antipsychotics is sparse. A large clinical trial on the impact of pharmacogenetic interventions for 26 drugs of different medical areas did not find clear benefits, although a reduction in side effects on the subgroup of patients treated with antipsychotic and antidepressant medications was observed [87]. A strategy including gathering of clinical and environmental information, study of pharmacological interactions, pharmacogenetic characterisation of the patients and adjusting the pharmacological treatment according to the information obtained has given good results in patients resistant to treatment with neuroleptics [88]. Pre-emptive strategies can also be clinically beneficial. It has been estimated that pre-emptive pharmacogenetic testing could be useful to predict 95% of vulnerable patients’ exposure to inadequate drugs [89]. Routinary pharmacogenetic testing of patients that require treatment with antipsychotics may significantly decrease the prevalence of side effects, as shown in our previous study [4]. Pharmacogenetic interventions for the selection of antipsychotic treatment results in higher improvement in comparison with treatment as usual [90]. Pharmacogenetic testing increases treatment adherence, leading to greater efficacy [91]. However, further evidence in different clinical settings is required to confirm the clinical utility of genetic testing for antipsychotics.

Emerging evidence illustrates the cost-effectiveness of the implementation of pharmacogenetics in psychiatry. Pharmacogenetic-guided antidepressant treatment resulted in significant savings in medication and clinical care [92,93]. CYP2D6 genotyping is cost-effective in patients treated with neuroleptics and antidepressants [91,94,95,96]. The combination of clinical, environmental and pharmacogenetic information for the personalisation of treatment in resistant patients achieved a cost–benefit ratio of 3.31–3.59 with a reduction of direct and total costs in most patients [95]. The cost-effectiveness of pharmacogenetic testing to prevent clozapine-induced agranulocytosis has been proven but only in patients with HLA susceptibility alleles [97]. Rapid and low-cost pharmacogenetic interventions in treatment-resistant autistic patients resulted in a reduction in hospital stays and clinical visits [80]. Further evidence of the cost-effectiveness of pharmacogenetic interventions will facilitate their implementation in national health services.

A significant barrier is the existence of pharmacogenetic guidelines for the adjustment of only a limited number of antipsychotics. The Dutch Pharmacogenetics Working Group (DPWG) has recently issued guidelines for the adjustment of aripiprazole, brexpiprazole, haloperidol, pirmozide, risperidone, zuclopenthixol and quetiapine according to CYP2D6 and CYP3A4 polymorphisms [5]. However, they conclude that no useful pharmacogenetic information is available for the commonly used antipsychotics clozapine and olanzapine. In a previous study, we used local clinical guidelines for the adjustment of doses of several FGA and SGA antipsychotics [4]. Dose adjustment according to CYP2D6- predicted metabolising rates resulted in a significant reduction of the toxicity induced by the antipsychotics aripiprazole, haloperidol and risperidone, confirming the utility of the existing guidelines. Furthermore, the adjustment of clozapine and olanzapine according to CYP1A2 and CYP2C19 polymorphisms also resulted in a reduction of side effects and improved efficacy in treatment-resistant patients [80]. Further research to provide evidence and specific guidelines for clozapine and risperidone is required.

Long waits for test results can diminish the clinical utility of pharmacogenetics. Commercial pharmacogenetic tests may take a minimum of 2–3 weeks to provide results. Polygenic risk scores may predict treatment response with a higher level of accuracy [72]. However, the increase in genotyping expense and in the time until results delivery significantly diminishes clinical utility. Biochemical measures (determination of metabolite plasma levels) obtained after a few weeks from the start of the treatment can provide more accurate information on drug clearance as the effect of concomitant treatments, diet and other environmental factors are reflected. Nevertheless, although genetic prediction of antipsychotic response may not achieve 100% accuracy, pharmacogenetic interventions for dose adjustment can be highly beneficial when applied at the start of the treatment, thus allowing for the selection of the right dose or alternative treatments. Rapid pharmacogenetic interventions that provide clinical recommendations within 24–48 h of request are of significant clinical utility [2,80]. The genotyping of key polymorphisms in CYP enzymes can be easily performed in most clinical laboratories and produce information at the start of the treatment, facilitating personalisation and increasing clinical value.

## 7. Discussion

As described in previous sections, the information on functional variants in CYP enzymes for the adjustment of antipsychotic clinical doses has proven to be of significant clinical utility and should be incorporated as a prescription tool. Furthermore, the genotyping of genetic variants in CYP1A2, CYP2C19, CYP2D6 and CYP3A4 for the adjustment of the dose of commonly used antipsychotics can be cost-effective. However, given the variability of genotype and clinical recommendations provided by pharmacogenetic tests [98], a standardisation of the CYP markers investigated and the pharmacogenetic-guided recommendations should be performed. Currently available genetic tests interrogate a variety of CYP genes and variants, providing different information and clinical recommendations that should be standardised. In cases where sequencing technologies to detect novel or known functional variants are not available, a list of CYP pharmacogenetic markers useful to determine the metabolic status of patients of different ethnicities should be compiled. Additionally, the clinical recommendations based on pharmacogenetic information should also be standardised. Detailed information on both key CYP genetic markers and relevant dose changes for antidepressants and antipsychotics have been issued by the CPIC and DWPG consortiums [5,76,77]. Both organisations compile clinical, pharmacokinetic and pharmacogenetic information to provide precise information on required dose changes, and their recommendations should be followed to elaborate reports on pharmacogenetic-guided clinical recommendations. Alternative recommendations with insufficient confirmation should be avoided. Standardised pharmacogenetic tests and recommendations will increase their reliability and facilitate their understanding and interpretation.

Finally, it is necessary to increase the education and training of clinical staff on the use and utilisation of pharmacogenetic information as a prescription tool for antipsychotic medications, either as a help in treatment-resistant patients or as a routinary pre-emptive prescription tool to increase the efficacy and safety of treatments. Although the teaching of pharmacogenetic strategies is being introduced in faculties in several countries, further education should be provided at different stages of training and in clinical settings to facilitate the use of pharmacogenetics for the personalisation of antipsychotic treatment.

## 8. Conclusions

Pharmacogenetic and pharmacogenomic research have helped to unravel the mechanism of action of antipsychotics and to identify biomarkers of response. However, most biomarkers have modest clinical utility, as their capacity to discriminate between different antipsychotics is reduced. Functional biomarkers in CYP metabolic enzymes are of greater utility as they can be used to adjust clinical doses of antipsychotics, resulting in greater efficacy and safety. Nevertheless, the implementation of pharmacogenetic interventions in psychiatry is minimal. Further evidence of the clinical benefits and cost-effectiveness, rapid and low-cost pharmacogenetic tests and specific pharmacogenetic guidelines for antipsychotics are required to increase their implementation. It is envisaged that artificial intelligence combining information from different areas (genetic, epigenetic, clinical, pharmacological and demographic) will produce a much more accurate prediction of response and will further the personalisation of antipsychotic treatment. However, given the complexity of such an approach combining information on multiple factors that need to be measured before the start of the treatment and the high cost that it will imply, it may be years until it is practicable in clinical settings.

**Table 1:** Summary of candidate-gene studies reporting significant associations.

Gene	Variant	Drug	*n*	Association	Ref.
31 genes	202 SNPs	SGA	113 Caucasians	Four SNPs in *DRD2*, *SLC18A2*, *HTR2A* and *GRIK3* contributed significantly to the risk of side effects (*p* = 1 × 10^−4^).	[99]
38 genes	several	SGA	300 Caucasian	Nominally significant association between antipsychotic dosage and *GFRA1* variants.	[100]
380 genes	several	SGA and FGA	240 several ethnicities	*NALCN* rs2152324 had most significant association with response (*p* = 0.004). Not significant after FDR correction.	[101]
74 genes	several	several	279 Caucasians	*BDNF* significantly associated with treatment resistance: rs11030104 (OR = 2.57), rs10501087 (OR = 2.19) and rs6265 (OR = 2.08)	[23]
*ADRB2*, *DRD3* and *SLC6A4*	several	Risperidone	111 Caucasians	Allele 16Gly of *ADRB2* significantly associated with higher risk of sexual adverse events (*p* = 0.002)	[102]
*BDNF*	4 SNPs	Clozapine	257 Caucasians	rs11030104 and Val66Met associated with response (*p* = 0.04; 0.007, respectively). rs1519480 associated with WG (*p* = 0.04).	[22]
*C4A* and *C4B*	several	FGA	87 Caucasians	Number of copies of *C4*BL nominally associated with TD severity (*p* = 0.020)	[103]
*CNR1*, *FTO*, *MC4R*, *LEP* and *FAAH*	several	Risperidone	225 Caucasians	Variants in *CNR1* (*p* = 1 × 10^−5^) and *LEP* (*p* = 1.4 × 10^−4^) associated with AIWG	[41]
*COMT* and *DRD2*	several	Risperidone	690 Chinese	*COMT* rs4680, *DRD2* rs6275, rs1801028 and rs6277 associated with PANSS improvement (*p* = 0.05)	[19]
*COMT*	rs4680 and rs4818	SGA	521 Caucasians	rs4680 A allele and rs4680–rs4818 C-A haplotype associated with olanzapine response, but not with response to other antipsychotics	[20]
*CYP1A2* and *CYP2D6*	several	FGA or Risperidone	475 Caucasians	CYP1A2*1F & CY2D6*4 associated with TD in patients on antipsychotics for a long time (*p* = 0.03)	[27]
*CYP1A2* and *CYP2B6*	several	Aripiprazol	19 Caucasians	CYP1A2 UM & CYP2B6*1/*1 associated with aripiprazol- induced side effects	[104]
*CYP2C19*, *LEPR*, *CYP1A2*, *HTR2C* and *ABCB1*	several	Clozapine	60 Caucasians	Clozapine levels in patients with metabolic syndrome were significantly higher compared to those without (*p* < 0.01) and were associated with CYP2C19*2 (*p* = 0.04)	[25]
*CYP2D6*	several	FGA and SGA	198 Caucasians	Individuals with either increased or no CYP2D6 activity were at higher risk of having TD	[28]
*CYP2D6*	several	Risperidone	257 several ethnicities	Children and adolescents with PM variants showed poorer response to risperidone treatment	[29]
*CYP2D6*	*4	Haloperidol	150 Caucasians	Carriers of *4 variant presented worse safety profile (*p* < 0.001)	[30]
*CYP3A4*	several	Olanzapine	CATIE sample	rs472660 significantly predicted olanzapine clearance (*p* = 5.9 × 10^−7^).	[105]
*DISC1*	several	FGA or SGA	193 Caucasians	Two SNPs nominally associated with TD severity (*p* < 0.05).	[106]
*DRD1*	rs4532	several	124 Brazilians	G-allele associated with treatment resistance (*p* = 0.001; adjusted OR = 2.71). GG had five-fold risk compared to A (*p* = 0.010; OR = 5.56).	[107]
*DRD2*, *DRD3*, *HTR2A*, *HTR2C*, *COMT*, *NQO1*, *RGS2* and *MnSOD*	13 SNPs	not specified	402 Dutch	*DRD2* TaqI associated with akathisia (OR = 2.3, *p* = 0.001), *DRD2* −141C associated with TD (OR = 0.20, *p* = 0.001)	[32]
*DRD2*	rs2514218	Haloperidol and Risperidone	100 Americans	In the aripiprazole group, C/C homozygotes had more akathisia; in the risperidone group, male T allele carriers had greater prolactin elevations	[37]
*DRD2* and *DRD3*	rs1800497, rs6277 and rs6280	Cariprazine	20 Caucasians	DRD2 rs1800497 and rs6277 associated with cariprazine response	[9]
*DRD2* and *DRD3*	several	SGA	129 Caucasians	DRD2 rs1799732, DRD3 rs6280, and HTR2A rs7997012 associated with treatment resistance.	[10]
*DRD3*, *DRD2*, *HTR2A*, *HTR2C*, *COMT* and *MTHFR*	several	several	329 Caucasians	*DRD3* 9Gly and *MTHFR* 677-T had better response (*p* = 0.034 and *p* = 0.019, respectively).	[7]
*DRD4*, *HTR2A*, *TPH1*, *SLC18A1* and *COMT*	several	Haloperidol	198 Tartars	Several associations of *DRD4*, *HTR2A*, *TPH1* and *SLC18A1* polymorphisms with antipsychotic response	[8]
*EP300*	expression levels	several	226 Caucasians	EP300 expression levels significantly associated with increases in BMIR, cholesterol levels and triglyceride concentrations	[108]
*FKBP5*, *NR3C1*, *BDNF* and *NTRK2*	several	Clozapine	591 Caucasians	Several associations between *FKBP5* rs1360780, *NTRK2* rs1778929 and rs10465180 with response	[109]
*FTO*	several	SGA	259 + 91 Caucasians	In a subpopulation without additional weight-inducing comedication (*n* = 178), rs7185735-G carriers gained 3.4 times more weight (1.69 ± 3.1 kg, *p* = 0.019)	[110]
*GLP1R*	several	SGA	464 Caucasians	Haplotypes associated with response to olanzapine (*p* = 0.002), perphenazine (*p* = 0.01), quetiapine (*p* = 0.008), risperidone (*p* = 0.02) and ziprasidone (*p* = 0.007)	[111]
*GRIN2A*, *DRD3*, *HTR2C*, *DRD4* and *GRIN2B*	42 SNPs	FGA or SGA	431 + 168 Caucasians	Several significant associations with TD were identified, but only GRIN2A (rs1345423) was found in both patient populations	[112]
*GRM3*	rs1468412	Risperidone	61 Caucasians	*GRM3* rs1468412 associated with worsening spatial working	[113]
*HLA*	several	Clozapine	180 neutropenia/ 1396 controls	*HLA-DQB1* rs113332994 associated with clozapine-induced agranulocytosis (OR = 16.31)	[45]
*HRH1* and *CHRM3*	several	Several	430 Caucasians	*HRH1* haplotype rs346074–rs346070 associated with BMI (*p* = 0.025) and obesity (*p* = 0.005) in patients using high-H1 affinity antipsychotics	[15]
*HRH3*	several	Risperidone	129 Han Chinese	rs3787429 (*p* = 0.013–0.087) and rs3787430 (*p* = 0.024–0.010) associated with efficacy after 4–8 weeks, respectively	[16]
*HRH4*	5 SNPs	Risperidone	113 Han Chinese	rs4483927 TT genotype predicts poor therapeutic response on the positive, negative, general and total scales of PANSS scores (*p* = 0.017, 0.019, 0.021 and 0.002, respectively)	[17]
*HSD11B1*	several	SGA	478 Caucasians	*HSD11B1 rs846910-A*, *rs375319-A* and *rs4844488-G* allele carriers associated with lower BMI in women	[114]
*HTR2A*	rs6313	Olanzapine or Risperidone	221 Caucasians	T allele carriers showed better response than non-carriers	[12]
*HTR2C*	−759T/C	SGA	48 female Caucasians	T allele carriers gained less weight as compared to patients who did not have the allele	[36]
*HTR2C*	−759T/C	Risperidone	108 Thai	5-HT2C -759-T/C associated with hypertension but not with WG	[39]
*HTR3A*	rs1062613 and rs2276302	several	101 Indian patients	rs1062613-T and rs2276302-G alleles significantly associated with good clinical response to clozapine (*p* = 0.02)	[13]
*HTR7*	several	Aripiprazol	100 Japanese	rs12412496-rs7916403-rs1935349 A-T-A haplotype correlated with worse improvement in the cognition score (*p* = 0.046).	[115]
*LEP* and *LEPR*	several	FGA or SGA	181 Caucasians	Significant association between a *LEP* haplotype (rs7799039G–rs10954173G–rs3828942G) and AIWG (*p* = 0.035)	[40]
*MAOA*, *MAOB*, *DRD1*, *DRD2*, *DRD3*, *DRD4* and *SLC6A3*	41 SNPs	FGA or SGA	446 Caucasians	Association between *MAOB* rs1799836 and HPRL in men. *SLC6A3* rs40184 and rs3863145 associated with HPRL in risperidone/paliperidone subgroup	[116]
*MC4R*	rs489693	SGA 4 weeks	341 Caucasians	rs489693 A/A carriers showed 2.2 times higher weight increase than carriers of the C/C genotype (*p* = 0.039)	[43]
*MC4R*	rs17782313	SGA 4 weeks	51 Caucasians	rs17782313 C/C carriers higher risk of WG and BMI increase, with a dose effect of the C-allele (*p* = 0.002).	[117]
*MC4R*	rs489693 and rs17782313	FGA or SGA	1991 Chinese	Recessive effects of rs489693 on AIWG, WC and triglyceride change %, with A/A incurring more metabolic adverse effects	[44]
*MC4R*	rs17782313	Amisulpride and Olanzapine	212 Several	C carriers had higher WG than T homozygotes	[35]
*NEUROD2*	several	SGA	167 Caucasians	rs11078918 and rs12453682 associated with change in neuropsychological test results (*p* = 0.02–0.001).	[118]
*NOS1AP*	rs1214382 and rs10494366	not specified	347 Caucasian	rs12143842-CC and rs10494366-TT male carriers show positive correlation of QTc length with antipsychotic dosage	[119]
*NPY5R*	several	FGA and SGA	99 Russians	rs11100494- C predisposes to AIWG (OR = 33.48, *p*< 0.001)	[120]
*OXTR*, *CNR1*, *DDC* and *DRD2*	several	Clozapine or SGA	196 Chileans	*OXTR* rs2228485, *CNR1* rs806368 and rs1049353, and *DDC* rs10499696 associated with treatment resistance (*p* by genotype: 0.02, 0.001, 0.001 and 0.0003, respectively)	[121]
*PLEKHA6*	rs7513240, rs4951353	not specified	263 Caucasians	rs7513240 and rs4951353 (A/G) associated with therapy response with different PANSS improvement after 4 weeks	[122]
*PRKAR2B*	16 SNPs	Clozapine and Olanzapine	99 Caucasians	rs9656135 minor allele carriers higher weight increase during treatment.	[123]
*PTPRD*	4 SNPs	Clozapine or Olanzapine	201 Caucasians and Africans	rs73398242 associated with AIWG in Europeans (*p* = 0.002) and with rs13294608 in African Americans (*p* = 0.003).	[124]
*RELN*	15 SNPS	SGA	260 Chinese	Two SNPs associated with antipsychotic treatment response (rs155333, *p* = 0.010 and rs6465938, *p* = 0.049)	[125]
*RGS2*	several	Haloperidol	258 Russians	RGS2*T/*T (rs2746073), *C/*C (rs4606) and *A/*A (rs2746071) associated with increased risk of antipsychotic-induced Parkinsonism	[126]
*SLC18A2*	9 SNPs	FGA long-term	217 Caucasians	rs2015586 and rs363224 SNPs associated with TD and AIMS scores.	[127]
*SLC6A5*, *GAD1*, *GRIA1*, *GRIA3*, *GRIA4*, *GRID2*, *GRIK1*, *GRIK2*, *GRIK3*, *GRIK4*, *GRIN2B*, *GRM1* and *GRM4*	62 SNPs	several	101 + 71 + 118 Caucasian patients	SLC6A5 rs2298826 associated with a rapid rise of motor side effects at the beginning of the treatment (*p* = 0.0002)	[128]
*SNAP25*	several	SGA and FGA	3243 Chinese	rs6039769 significantly associated with AIWG (*p* < 0.001).	[129]
*SULT4A1*	rs2285162 and rs2285167	Olanzapine	87 Caucasians	rs2285162 [A]-rs2285167 [G] haplotype superior olanzapine response (*p* = 0.004) and less AIWG per month (*p* = 0.04)	[130]
*SV2C*	106 SNPs	SGA	466 Caucasians	rs11960832-T/T significantly worse response to olanzapine treatment (*p* = 2.94 × 10^−5^; FDR = 2.18 × 10^−2^)	[131]
*UGT1A4*, *UGT1A4* and *ABCB1*	7 SNPs	Olanzapine	91 Japanese	Sympathetic nervous activity higher in individuals with the *UGT1A4* rs2011425 G allele (*p* = 0.001).	[132]

**Abbreviations:** AIWG: antipsychotic-induced weight -gain; BMI: body mass index; FGA: first-generation antipsychotics; SGA: second-generation antipsychotics; SNP: single nucleotide polymorphism; TD: tardive dyskinesia; WC: waist circumference; WG: weight gain.

**Table 2:** Summary of genomic studies on antipsychotic medications.

Strategy	*n*	Treatment	Association	Ref.
GWAS	122 + 174 Japanese	several	Association DPP6 rs6977820 with antipsychotic-induced TD (*p* = 0.008)	[55]
GWAS	96 + 169 Caucasians	FGA or SGA	Two SNPs (rs7912580 and rs2412459) associated with response in both samples, located between ARID5B and RTKN2 genes	[48]
GWAS and WES	163 Caucasians	Clozapine	*HLA-DQB1* (126Q) (*p* = 4.7 × 10^−14^, OR = 0.19) and HLA-B (158T) (*p* = 6.4 × 10^−10^, OR = 3.3) associated with clozapine-induced agranulocytosis	[46]
Array 1995 genes	89 Caucasians	Olanzapine or Risperidone	Significant associations between treatment response and SNPs in the chromosome 6, where the human leukocyte antigen (HLA) is located	[49]
GWAS	189 + 86 Caucasians	SGA	*OGFRL1* rs9346455 significantly associated with AIWG (*p* = 0.005)	[57]
WES	11 + 103 + 87 several ethnicities	FGA or SGA	rs13025959 in *MYO7B* (E1647D) and rs10380 in *MTRR* (H622Y) associated with antipsychotic response	[51]
GWAS	742 Indians	FGA or SGA	*CCL2* rs4795893 (*p* = 7.62 × 10^−4^) and rs4586 (*p* = 1.13 × 10^−3^), *GRIA4* rs2513265 (*p* = 1.44 × 10^−3^), *ADCY2* rs1544938 (*p* = 7.68 × 10^−4^), and *NRG1* rs13250975 (*p* = 6.81 × 10^−3^) and rs17716295 (*p* = 8.71 × 10^−3^) associated with response	[50]
GWAS	534 + 547 Chinese	SGA	*PTPRD* rs10977144 (*p* = 9.26 × 10^−9^) and rs10977154 (*p* = 4.53 × 10^−8^), and *GFPT2* rs12386481 (*p* = 1.98 × 10^−7^) associated with AIWG	[58]
GWAS	50 + 380 Japanese	Clozapine	Variants in the human leukocyte antigen (HLA) region (rs1800625, *p* = 3.46 × 10^−9^, OR = 3.8) associated with agranulocytosis	[47]
WES	316 + 1920 Chinese	FGA or SGA	Rare genetic variants in *NMDA* and *AMPA* enriched in the non-responder group	[52]
WES	82 Jewish	not specified	*RIMS2* showed significant enrichment of qualifying variants in TD patients (*n* = 39) (*p* = 5.32 × 10^−8^)	[56]
GWAS	552 African ancestry	Clozapine	*ACKR1* rs2814778-C/C carriers more likely to develop neutropenia and have to stop clozapine treatment (OR = 20.4, *p* = 3.44 × 10^−7^)	[60]
Sequencing 143 genes	79 + 159 Han Chinese	Olanzapine	rs324026 (*p* = 0.023) and rs12610827 (*p* = 0.043) associated with response	[54]
GWAS	339 several ethnicities	Amisulpride	Significant association in a locus not previously associated with AIWG (rs78310016; *p* = 3.66 × 10^−8^). Minor allale carriers had an OR of 3.98 (*p* = 1 × 10^−3^) for AIWG	[59]
GWAS	2040 Chinese	FGA or SGA	*ATAD3B* rs20005072 and *SKIL* rs186507741 associated with antipsychotic-induced QTc interval change.	[61]
GWAS and WES	189 + 222 Chinese	Risperidone	GWAS revealed a significant association between *GRM7* SNPs (rs141134664, rs57521140 and rs73809055) and treatment response	[53]

**Abbreviations:** AIWG: antipsychotic-induced weight -gain; FGA: first-generation antipsychotics; GWAS: genome-wide association study; SGA: second-generation antipsychotics; SNP: single nucleotide polymorphism; TD: tardive dyskinesia; WES: whole exome sequencing; WG: weight gain.

### Summative Paragraph

- Genetic variants in genes coding for drug targets -dopamine and serotonin receptors in particular- may influence the efficacy and safety of antipsychotic medications.
- Functional variants in CYPs are associated with antipsychotic availability.
- Dose adjustment according to CYP functional variants present may help to improve adherence, efficacy and safety of antipsychotics.
- Clinical implementation of pharmacogenetic interventions for personalisation of antipsychotic treatment is limited.
- Improved clinical guidelines based on pharmacogenetic data, education and training in pharmacogenetics, reduced costs and shorter delivery times may increase implementation.
- Further research on the combined effect of pharmacogenetics, phenoconversion, and clinical and environmental factors is required.

## Data Availability

Not applicable.
